# Comparative performance of biomarkers of alcohol consumption in a population sample of working-aged men in Russia: the Izhevsk Family Study

**DOI:** 10.1111/add.12251

**Published:** 2013-07-05

**Authors:** Helen McDonald, Svetlana Borinskya, Nikolay Kiryanov, Artyom Gil, Anders Helander, David A Leon

**Affiliations:** London School of Hygiene and Tropical MedicineLondon, UK1; Vavilov Institute of General GeneticsMoscow, Russia2; Izhevsk State Medical AcademyIzhevsk, Russia3; First Moscow State Medical UniversityMoscow, Russia4; Department of Laboratory Medicine, Karolinska InstituteStockholm, Sweden5

**Keywords:** Alcohol drinking/blood, biological markers, male, Russia/epidemiology

## Abstract

**Aims:**

To assess the performance of a range of biomarkers of alcohol consumption in a heavy-drinking population of working-aged Russian men.

**Design:**

Cross-sectional study of men originally sampled at random from a population register.

**Setting:**

Izhevsk, a Russian city with a population of 650 000 people.

**Participants:**

A total of 1023 men aged 27–59 years living in Izhevsk who took part in a health check examination in 2008–2009.

**Measurements:**

Self-reported alcohol consumption, hazardous drinking behaviours, socio-economic position, anthropometric measurements plus blood levels of alcohol biomarkers [carbohydrate-deficient transferrin (CDT, gamma-glutamyl transferase (GGT), alanine aminotransferase (ALT), aspartate aminotransferase (AST) and mean cell volume of erythrocytes (MCV)] and hepatitis B and C status.

**Findings:**

In the year before interview there was a high prevalence of high-risk alcohol consumption indicated by consumption of non-beverage alcohols (5%), problem drinking behaviours (4.4%) and alcohol consumption exceeding an average 40 g per day (12.6%). All biomarkers were associated strongly with total beverage alcohol consumption even after adjustment for confounders. CDT performed best as an alcohol biomarker, with a sensitivity of 67% and specificity of 71% for detecting an average consumption of more than 40 g per day versus less. For all biomarkers sensitivity was considerably lower than specificity. Hazardous drinking patterns *per se* were not well detected by any of the biomarkers, all with sensitivity below 60%.

**Conclusions:**

In a Russian population with high levels of alcohol consumption, carbohydrate-deficient transferrin (CDT) might be the most sensitive and specific biomarker for detecting ethanol consumption above 40 g/day. A biomarker reflecting hazardous drinking patterns has yet to be established.

## INTRODUCTION

Between 1990 and 1994, male life expectancy at birth in Russia fell by 6 years. Since then it has undergone a series of sharp fluctuations mainly driven by changes in the mortality of working-aged men, which are believed to be principally the result of changes in alcohol consumption [1]. Since 2005 life expectancy has increased steadily, but remains appreciably lower than that of other European countries [2].

Estimates of adult alcohol consumption in Russia range from 15.5 to 18.5 l per capita [3–5]. Even the lowest of these estimates is among the highest in Europe [3]. The pattern of alcohol consumption in Russian men is distinguished by a preference for highly concentrated alcohol, much from illicit sources, consumed in intense bouts [3]. Such patterns of irregular heavy drinking have detrimental effects on health, independently of the total amount consumed, and might be associated with additional mortality [3,6,7]. Using a variety of different approaches, it has been estimated that alcohol accounts for between a third and a half of all deaths among working-aged Russian men [1,8–10].

Self-reports of alcohol consumption have inherent and well-recognized limitations. This is particularly true in Russia, as an appreciable proportion of alcohol intake (estimated between a third and a half) is from unrecorded sources such as homebrews and ‘surrogates’ (non-beverage alcohols such as aftershave and medicinal tinctures) [1,4,5]. Surrogate alcohols contain variable but high concentrations of 60–90% ethanol, meaning that quantification of ethanol intake is difficult due to lack of standardization of concentration and ‘drink’ size [11–13]. In this context, biomarkers of alcohol consumption can provide a further, independent, source of information about drinking behaviour.

The most common alcohol biomarkers employed so far include the liver enzymes gamma-glutamyl transferase (GGT), alanine aminotransferase (ALT) and aspartate aminotransferase (AST), the mean cell volume of erythrocytes (MCV) and carbohydrate-deficient transferrin (CDT), all measured in blood [14,15].

Population-based studies of the relationship between alcohol consumption and these biomarkers have been based largely in western Europe, Scandinavia, the United States and Canada, and found weak relationships [16,17]. Systematic reviews have been hindered by varying study designs, subject characteristics, assay methods, test cut-offs and drinking thresholds, but CDT appears to be consistently more specific than GGT, and there may be value in combining the two biomarkers [15,18,19].

Alcohol biomarkers have been employed rarely in epidemiological studies in Russia. One study compared self-reported alcohol consumption, GGT and CDT levels in the neighbouring populations of Karelia, Finland and Karelia, Russia [20]. For similar reported levels of alcohol consumption, the proportion of men with raised GGT levels was higher in Finland than Russia, while the proportion with elevated CDT levels was almost four times higher in Russian compared to Finnish men.

Given the distinctive and hazardous pattern of alcohol consumption found in Russia, and the paucity of relevant studies from Russia, there is a need to assess whether these common alcohol biomarkers are associated with self-reported alcohol consumption and behave in a similar way as elsewhere. This is an important prerequisite for developing their wider use in Russia, where alcohol remains such a determinant of poor health and mortality.

In this paper we present the results of a cross-sectional, population-based study of working-aged men resident in the Russian city of Izhevsk. This is the first study to include an assessment of hazardous drinking patterns and non-beverage alcohol use, and to compare the performance of all standard alcohol biomarkers.

## MATERIALS AND METHODS

### Study population

The study was conducted in 2008–2009 in the Russian city of Izhevsk. This city has a population of 650 000, with a typical demographic profile for a medium-sized Russian city, although it has a relatively high suicide rate [12,21]. Participants were working-aged men who had been recruited originally at random for the Izhevsk Family Study in 2003–2006 from a population register, the majority of whom had been used as live controls in a case–control study of premature mortality [22]. Because the age structure of the controls was matched to that of the deaths in this original study, the age structure of the sample is older than that of the city population. Of the 2041 men recruited originally, 1515 men were re-interviewed in 2008–2009. The cohort was restricted to participants with at least one biomarker result in 2008–2009.

Interviews were conducted face to face by a team of Russian sociologists using a questionnaire designed to collect very detailed information on alcohol consumption and drinking behaviours, with a time window of the previous 12 months. The frequency of consumption of beer, wine and spirits, together with the usual quantity per sitting, was combined with standard beverage strengths (beer 4%, wine 12%, spirits 43%) [23], to estimate the total consumption of beverage alcohol using the standard quantity–frequency approach [24]. A variable for behaviour indicating hazardous drinking was positive if any of the following were reported over the previous year: one or more episodes of *zapoi* (intensive bouts of continuous drunkenness lasting 2 or more days) [1]; or occurrence at least twice a week of excessive drunkenness or hangover or sleeping with clothes on because drunk.

At the end of the main interview, participants were offered a health check that occurred typically 3–4 weeks later. These were conducted by Russian medical doctors, in clinic or the men's homes, depending on participant preference. Height, weight, waist and hip circumference were measured using standard protocols. Each anthropometric measurement and blood pressure was repeated three times at the health check, and a mean was calculated. Blood samples were taken at the health check and processed within 12 hours at the Republican Blood Transfusion Centre in Izhevsk. Blood was spun and then aliquoted. All alcohol biomarker assays other than CDT were undertaken on the fresh samples, while the remaining aliquots were frozen and stored at −80°C. ALT and AST were measured using the Humalyzer 2000 analyser (HUMAN) using the kinetic method of detection [25,26] and GGT by the kinetic colorimetric method [27]. Hepatitis test kits were obtained from Vector Best (Novosibirsk, Russia): hepatitis B assays (for hepatitis B surface antigen) used test kits D-0544, confirming positive results with kit D-0546. Hepatitis C assays (for anti-hepatitis C immunoglobulin) used kit D-0772, confirming positive results with kit D-0776 [28]. Aliquots of the serum samples were transferred to the Moscow Research and Practical Center on Addictions for measurement of CDT by capillary electrophoresis using the Sebia Capillarys-2 multi-capillary analyser (Norcross, GA, USA) [29]. The Sebia Capillarys-2 multi-capillary device measures the percentage of transferrin as the disialotransferrin isoform [30].

### Data analysis

Four types of alcohol exposure were considered: current drinking (compared to no current drinking); hazardous drinking patterns (behaviours indicating hazardous drinking patterns or non-beverage alcohol drinking, compared with all other participants, drinkers and non-drinkers combined); high-volume drinking (average more than 40 g/day over the previous year, compared with all other drinkers and non-drinkers combined); and risky drinking (either high-volume drinking or hazardous drinking patterns, compared with all other drinkers and non-drinkers combined). Analyses of hazardous, high-volume or risky drinking were restricted to participants with the relevant data describing their alcohol consumption.

Biomarkers were considered to be elevated if higher than the following standard thresholds: GGT 51 U/l, ALT 40 U/l, AST 37 U/l and MCV 100 fL [16]. The cut-off used for CDT depends on the measurement method, as different techniques have different analytical sensitivities [31]. The threshold for CDT in this study was 1.3%, as recommended by the assay manufacturer [30]. All analyses of MCV and CDT were restricted to participants with a result for the relevant biomarker.

The reference ranges of ‘normal’ biomarkers in this sample were defined as the exponentiated 95% range of each of the logarithmically transformed biomarkers, among alcohol drinkers drinking fewer than two litres of alcohol per year, with normal body mass index (BMI) and negative hepatitis B and C status.

Receiver operating characteristic (ROC) curves, and the sensitivity and specificity at standard thresholds for each biomarker, were calculated for each type of alcohol exposure. The adjusted attributable fraction of elevated biomarkers (according to standard thresholds) was calculated according to the Mantel–Haenszel approach [32].

To explore the associations between biomarker elevation and alcohol consumption, logistic regression and likelihood ratio tests were used. The binary outcome variable was defined according to whether an individual was in the top quintile of each biomarker to enable comparison between analysis of each biomarker and to ensure that each analysis had sufficient power. The data set was restricted to participants with the relevant biomarker result and no missing data for any confounders (age, BMI, waist : hip ratio, socio-economic status, education, hepatitis B or C status). Non-beverage alcohol drinkers were excluded from analyses of total volume of ethanol consumed, as it was not possible to estimate the ethanol consumed from these sources due to wide variation in concentration and bottle/container size.

As a *post-hoc* analysis, combinations of CDT and GGT were investigated. Using standard thresholds, the sensitivity and specificity were calculated for elevation of: both CDT and GGT; either CDT or GGT; and CDT among participants with an elevated GGT. Multivariable logistic regression of the relationship of each of these combined biomarker outcomes with the volume of alcohol consumed among alcohol drinkers (excluding non-beverage drinkers) was conducted using top quintiles of biomarkers to ensure reasonable power.

The data were managed and analysed using STATA version 11.

## RESULTS

**Figure 1:**
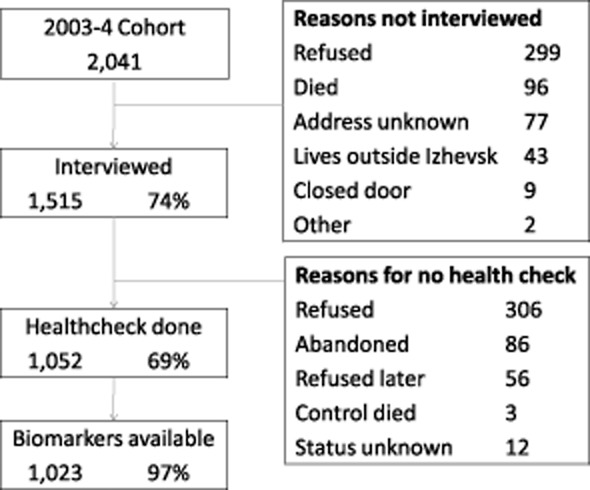
Study participation flow diagram

Of 2041 eligible men, 1515 were included in the sample, corresponding to a participation rate of 74.2%. Figure 1 illustrates the participation and loss to follow-up at each stage from the case–control study in 2003–2004 to inclusion in this analysis. At least three biomarkers (GGT, ALT and AST) were available for 1023 participants, 50.1% of those eligible. There was no evidence for any difference in frequency or volume of alcohol consumed between those who were and were not missing biomarkers (frequency *P* = 0.51; annual volume *P* = 0.99). Of the 1023 men with biomarkers available, two lacked both MCV and CDT results, and a further 26 lacked CDT results. Of the 1015 CDT assays performed, 18 were uninterpretable due to critical interference in the electropherogram [30].

The baseline characteristics of the 1023 participants are described in Table 1. Most of the non-drinkers were former drinkers, with only eight being life-long abstainers.

**Table 1:** Baseline characteristics of participants included in the analysis *n* = 1023.

	*Number*	*%*
Drink any beer, wine, spirits or surrogates		
Currently, or in the previous 12 months	888	86.8
Previously, but not within the previous 12 months	127	12.4
Never (life-long abstainer)	8	0.8
Frequency of consuming any alcohol in the previous 12 months		
Daily	21	2.1
5–6 times/week	64	6.3
3–4 times/week	135	13.2
1–2 times/week	341	33.3
1–3 times/month	326	31.9
Never or almost never	135	13.2
Missing	1	0.1
Total amount of ethanol from beverages per year (l/year) in the previous 12 months		
Non-beverage drinker	135	13.2
<2	189	18.5
2–4	206	20.1
5–9	194	19.0
10–19	166	16.2
20 or more	120	11.7
Missing	13	1.3
Age at interview (years)		
27–34	87	8.5
35–39	98	9.6
40–44	119	11.6
45–49	198	19.4
50–54	252	24.6
55–59	269	26.3
Socio-economic status		
Neither car nor central heating	61	6.0
Car or central heating	473	46.2
Both car and central heating	489	47.8
Education		
Incomplete secondary or lower	45	4.4
Secondary	748	73.1
Higher than secondary	230	22.5
Body mass index, BMI (kg/m^2^)		
Normal weight <25	426	41.6
Overweight 25–29	406	39.7
Obese 30–34	146	14.3
Severely obese ≥35	39	3.8
Missing	6	0.6
Waist: hip ratio (quartiles)		
Lowest quartile	257	25.1
Second lowest quartile	259	25.3
Second highest quartile	256	25.0
Highest quartile	249	24.3
Missing	2	0.2
Hepatitis B status (surface antigen)		
Negative	909	88.9
Positive	39	3.8
Inconclusive	19	1.9
Missing	56	5.5
Hepatitis C status (antibody)		
Negative	930	90.9
Positive	35	3.4
Inconclusive	2	0.2
Missing	56	5.5

GGT, ALT, AST and CDT levels were each elevated in between a sixth and a third of the sample, but very few (4%) had elevated MCV levels (Table 2). Even among the lowest category drinkers with normal BMI and negative hepatitis B and C status, the 95% reference range included participants with elevated GGT, AST and CDT levels. With a higher threshold of 80 U/l used in a previous study of a Russian population [20], the prevalence of elevated GGT was 11.3% (116 of 1023).

**Table 2:** Distributions of biomarkers in the sample population, *n* = 1023

*Biomarker*	*n*	*Geometric mean (95% confidence interval)*	*Lower limit of top quintile*	*Standard threshold used*	*Above standard thresholdn (%)*	*Sample 95% reference range^a^ (n)*
GGT	1023	33.3 (31.8–34.9)	53 U/l	51 U/l [16]	219 (21.4)	12.1–86.7 (57)
AST	1023	31.0 (30.1–31.9)	39.3 U/l	37 U/l [16]	238 (23.3)	18.7–55.7 (57)
ALT	1023	27.0 (26.1–27.9)	39.7U/l	41 U/l [16]	190 (18.6)	13.7–37.4 (57)
CDT	997	1.18 (1.12–1.26)	2.2%	1.3% [30]	334 (33.5)	0.40–3.50 (57)
MCV	1021	89.0 (88.7–89.4)	93.5 fL	100 fL [16]	37 (3.6)	71.9–98.0 (56)

a Among men with body mass index (BMI) <25 drinking over zero and fewer than 2 l of alcohol per year and with negative hepatitis B and C status. GGT = gamma-glutamyl transferase; AST = aspartate aminotransferase; ALT = alanine aminotransferase; CDT = carbohydrate-deficient transferrin; MCV = mean cell volume of erythrocytes.

### Biomarker sensitivity and specificity for detecting alcohol consumption

The performances of standard thresholds for biomarker elevation in detecting alcohol consumption are presented in Table 3. CDT had the highest sensitivity for detecting current drinking, hazardous drinking patterns and drinking more than an average 40 g alcohol per day, but these sensitivities were still poor. CDT was highly specific for current drinking, and the population-attributable fraction of elevated CDT was almost 100%. GGT also had a high specificity for current drinking, and the population-attributable fraction of elevated GGT from current drinking was also high. CDT was more sensitive to high-volume (>40 g/day) drinking than hazardous drinking patterns: ALT and AST were more sensitive to hazardous drinking patterns than volume. No individual biomarkers were more specific for detecting hazardous or high-volume drinking than current drinking.

**Table 3:** Sensitivity and specificity of standard biomarker thresholds for drinking patterns, and the attributable fraction of biomarker elevation to alcohol

	*Biomarker elevated*	*Sensitivity(%)*	*Specificity(%)*	*Exposure prevalence^a^*	*Adjusted OR^b^*	*PAF(%)^c^*
Gamma-glutamyltransferase (GGT)						
Current drinker	213/888	24.0	95.6	213/219	5.63	80.0
Not current	6/135					
Hazardous drinker	31/98	31.6	79.8	31/215	1.63	5.6
Not hazardous	184/910					
>40 g/day	38/127	29.9	80.1	38/214	1.47	5.7
<40 g/day	176/883					
Risky drinker	56/181	31.0	80.6	56/219	1.67	10.3
Not risky drinker	163/840					
Aspartate aminotransferase (AST)						
Current drinker	212/888	23.9	80.7	212/238	1.32	21.8
Not current	26/135					
Hazardous drinker	42/98	42.9	78.7	42/236	2.41	10.4
Not hazardous	194/910					
>40 g/day	44/127	34.7	78.4	44/235	1.58	6.9
<40 g/day	191/883					
Risky drinker	67/181	37.0	79.6	67/238	2.05	14.4
Not risky drinker	171/840					
Alanine aminotransferase (ALT)						
Current drinker	173/888	19.5	87.4	173/190	1.29	20.5
Not current	17/135					
Hazardous drinker	35/98	35.7	83.2	35/188	2.67	11.6
Not hazardous	153/910					
>40 g/day	37/127	29.1	83.0	37/187	1.87	9.2
<40 g/day	150/883					
Risky drinker	60/181	33.2	84.5	60/190	2.76	20.1
Not risky drinker	130/840					
Carbohydrate-deficient transferrin (CDT)						
Current drinker	333/866	38.5	99.2	333/334	106.84	98.8
Not current	1/131					
Hazardous drinker	52/94	55.3	69.1	52/326	2.53	9.6
Not hazardous	274/888					
>40 g/day	81/122	66.4	71.2	81/329	5.13	19.8
<40 g/day	248/862					
Risky drinker	102/175	58.3	71.8	102/333	3.53	22.0
Not risky drinker	231/820					
Mean cell volume (MCV)						
Current drinker	36/886	4.1	99.3	36/37	6.69	82.8
Not current	1/135					
Hazardous drinker	8/98	8.2	96.9	8/36	2.80	14.3
Not hazardous	28/908					
>40 g/day	11/126	8.7	97.2	11/36	3.82	22.6
<40 g/day	25/882					
Risky drinker	14/180	7.8	97.4	14/36	3.41	27.5
Not risky drinker	22/839					

a Prevalence of alcohol drinking pattern among those with elevated biomarkers.

b Odds ratio (OR) adjusted for age, body mass index, waist/hip ratio, socio-economic status, education and hepatitis B and C status.

c Population-attributable fraction (PAF).

The performance of biomarkers across a range of thresholds in detecting alcohol consumption are presented as ROC curves (Table 4). No biomarker performed notably better at detecting hazardous drinking patterns or volume than current drinking. The biomarkers with the largest difference from the performance expected by chance were CDT, GGT and MCV.

**Table 4:** Receiver operating characteristic (ROC) curves demonstrating the performance of biomarkers across a range of thresholds for detecting alcohol consumption

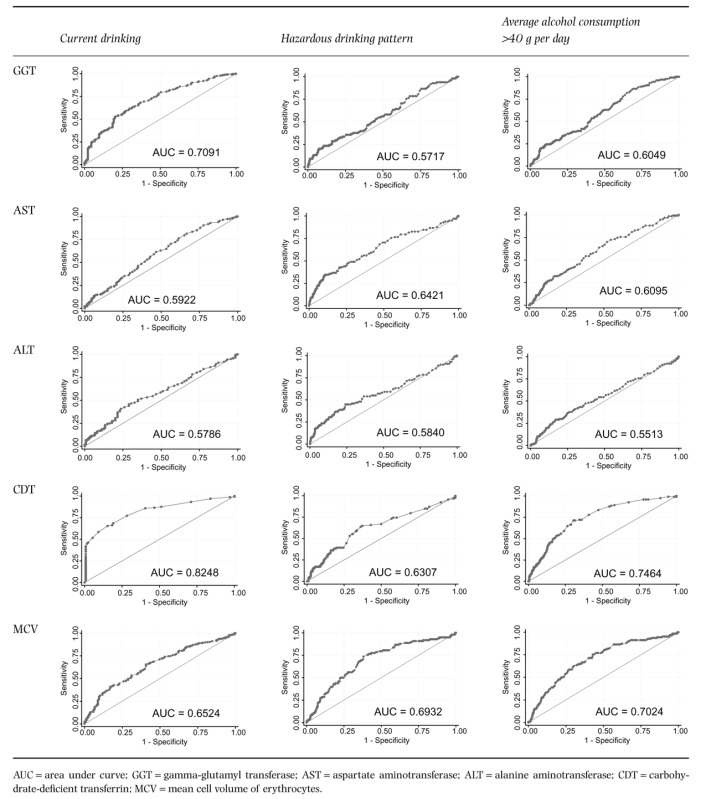

The performance of GGT and CDT combined for detecting alcohol consumption are presented in Table S2 (Supporting information). Elevation of either CDT or GGT had higher sensitivity than either biomarker alone, but at the cost of lower specificity. The performance of CDT as a confirmatory test among participants with elevated GGT is difficult to interpret due to small numbers.

### Logistic regression

The crude relationships between alcohol consumption in the previous 12 months and top quintile biomarker results are presented in Table 5. There was very strong evidence (*P* < 0.001) of a relationship between all biomarkers and frequency and volume of alcohol consumption.

**Table 5:** Univariable analysis of association of alcohol drinking with top quintile biomarkers

	*Number*	*GGT*		*AST*		*ALT*		*CDT*		*MCV*	
		*n*	*%*	*n*	*%*	*n*	*%*	*n*	*%*	*n*	*%*
Drink any beer, wine, spirits or surrogates (current; within the previous 12 months: previous; prior to the previous 12 months: never; life-long abstainer)											
Never	8	1	12.5	2	25.0	5	62.5	0/8	0	0/8	0
Previous	127	5	3.9	19	15.0	13	10.2	1/123	0.81	12/127	9.5
Current	888	201	22.6	189	21.3	188	21.2	205/866	23.7	196/886	22.1
*P* (χ^2^)		<0.001		0.24		<0.001		<0.001		0.001	
Frequency of consuming any alcohol in the previous 12 months											
(Almost) never	135	6	4.4	21	15.6	18	13.3	1/131	0.8	12/135	8.9
1–3 days/month	326	45	13.8	49	15.0	55	16.9	40/318	12.6	41/325	12.6
1–2 days/week	341	85	24.9	69	20.2	70	20.5	73/332	22.0	78/341	22.9
3–4 days/week	135	44	32.6	42	31.1	38	28.2	54/133	40.6	46/135	34.1
5–6 days/week	64	17	26.6	18	28.1	14	21.9	26/61	42.6	19/64	29.7
Daily	21	10	47.6	11	52.4	11	52.4	12/21	57.1	11/20	55.0
*P* (χ^2^)		<0.001		<0.001		<0.001		<0.001		<0.001	
Total amount of ethanol from beverages per year (l/year) in the previous 12 months											
None	135	6	4.4	21	15.6	18	13.3	1/131	0.8	12/135	8.9
>0 and <2	189	22	11.6	25	13.2	29	15.3	20/184	10.9	21/188	11.2
2–4	206	38	18.5	32	15.5	31	15.1	31/200	15.5	28/206	13.6
5–9	194	54	27.8	38	19.6	38	19.6	49/191	25.7	50/194	25.8
10–19	166	47	28.3	52	31.3	54	32.5	44/161	27.3	43/166	25.9
20 or more	120	35	29.2	39	32.5	33	27.5	57/117	48.7	49/119	41.2
*P* (χ^2^)		<0.001		<0.001		<0.001		<0.001		<0.001	

GGT = gamma-glutamyl transferase; AST = aspartate aminotransferase; ALT = alanine aminotransferase; CDT = carbohydrate-deficient transferrin; MCV = mean cell volume of erythrocytes.

The results of univariable analysis of the relationship of possible confounders with top quintile biomarkers are available in Table S1 (Supporting information).

Table 6 presents the age-adjusted odds ratios describing the association between volume of alcohol consumption and top quintile biomarkers among alcohol drinkers, excluding those who drank surrogates. Both before and after adjustment for obesity, socio-economic status, education and hepatitis B and C status, there was strong evidence that higher volumes of alcohol consumption are associated with increased odds of CDT, GGT, ALT, AST and MCV levels in the top quintile. The size of the associations were not notably altered by adjustment. There was strong evidence for a linear relationship between alcohol volume and top quintile CDT, GGT and AST, and some evidence for MCV.

**Table 6:** Odds ratios of biomarker being in the top quintile for the sample excluding surrogates

	*GGT*			*AST*			*ALT*			*CDT*			*MCV*		
	*n = 782*			*n = 782*			*n = 782*			*n = 765*			*n = 781*		
	n	OR^a^	95% CI^b^	n	OR^a^	95% CI^b^	n	OR^a^	95% CI^b^	n	OR^a^	95% CI^b^	n	OR^a^	95% CI^b^
Total amount of alcohol from beverages per year (l/year) among current drinkers, adjusted for age															
>0 and <2	171	1	–	171	1	–	171	1	–	167	1	–	170	1	–
2–4	189	1.72	0.94–3.16	189	1.31	0.70–2.45	189	1.02	0.56–1.84	184	1.43	0.74–2.67	189	1.68	0.88–8.18
5–9	179	3.09	1.73–5.54	179	1.90	1.04–3.46	179	1.34	0.75–2.39	176	3.11	1.69–5.72	179	2.92	1.58–5.38
10–19	145	2.97	1.62–5.45	145	3.00	1.65–5.44	145	2.26	1.28–3.98	142	2.94	1.55–5.55	145	3.07	1.63–5.80
20+	98	3.21	1.67–6.12	98	3.22	1.70–6.12	98	2.24	1.20–4.17	96	7.75	4.05–14.84	98	6.18	3.21–11.91
*P*^c^		0.0001			0.0002			0.004			<0.0001			<0.0001	
Total amount of alcohol from beverages per year (l/year) among current drinkers, adjusted for BMI, waist/hip ratio, age, socio-economic status, education, hepatitis B and C status															
>0 and <2		1	–		1	–		1	–		1	–		1	–
2–4		1.70	0.90–3.21		1.48	0.77–2.85		1.03	0.54–1.96		1.68	0.83–3.41		1.75	0.90–3.40
5–9		3.46	1.88–6.36		2.15	1.14–4.03		1.53	0.82–2.85		3.42	1.78–6.59		3.01	1.60–5.67
10–19		2.93	1.55–5.53		3.06	1.63–5.75		2.45	1.31–4.57		3.36	1.68–.69		3.15	1.63–6.08
20+		3.04	1.52–6.06		3.13	1.57–6.20		2.47	1.23–4.98		9.49	4.66–19.31		5.94	3.00–11.77
*P*^d^^c^		0.0001			0.012			0.006			<0.0001			<0.0001	
*P* (linearity)^d^		0.0005			0.01			0.06			0.002			0.06	

a Odds ratio (OR);

b 95% confidence interval (CI);

c likelihood ratio test (LRT) for total amount of alcohol;

d likelihood ratio test (LRT) for linearity. GGT = gamma-glutamyl transferase; AST = aspartate aminotransferase; ALT = alanine aminotransferase; CDT = carbohydrate-deficient transferrin; MCV = mean cell volume of erythrocytes.

When the regression analysis was repeated including surrogate drinkers, and also using standard thresholds for biomarkers to define the outcome, the odds ratios observed were very similar (not presented).

The odds ratios describing the associations between volume of alcohol consumption and combinations of top quintile GGT and CDT biomarkers among alcohol drinkers, excluding non-beverage drinkers, are presented in Table S3 (Supporting information). There was strong evidence for a linear relationship between alcohol volume and both CDT and GGT being in the top quintile, and between alcohol volume and either CDT or GGT being in the top quintile. There was no evidence of a relationship of top quintile CDT with alcohol volume among participants with GGT in the top quintile, which may be due to small numbers.

## DISCUSSION

In this sample of working-aged Russian men, there was a high prevalence of high-risk alcohol consumption indicated by non-beverage drinking (5%), problem drinking behaviours (4.4%) and alcohol consumption exceeding an average 40 g per day (12.6%). CDT had the highest sensitivity and specificity to detect current alcohol consumption at standard thresholds, followed by GGT. No biomarkers performed better at detecting hazardous drinking (in terms of behaviour or amount) than current drinking. For all biomarkers, sensitivity was considerably lower than specificity. All biomarkers were associated strongly with annual alcohol consumption, even after adjustment for confounding, and CDT had the greatest size of association.

The prevalence of raised GGT levels differed from those found in Karelia by Laatikainen *et al*. [20]. Using the same GGT threshold of 80U/l [20], the prevalence of elevated GGT in the Izhevsk sample (11.3%) was higher than that observed among men in Karelia (3.9% in Russia, 8.9% in Finland). The Izhevsk sample contained more men in older age groups, but even among the oldest age group in Russian Karelian men (55–64 years) the prevalence of elevated GGT was only 4.7%. GGT is also determined by factors other than alcohol consumption, including BMI, smoking, diabetes mellitus and hepatobiliary disorders, and the prevalences of these may have differed [15,33]. The prevalence of elevated CDT was similar to that found among men in Karelia (37%), but use of different CDT assays limit the comparison [20].

The relationships between average alcohol consumption and biomarkers were similar to those seen in other populations. A large cross-sectional study of the relationship between alcohol consumption and GGT, ALT, AST and MCV among a population-based sample of 8708 adults in the United States found that all biomarkers increased with higher alcohol consumption. The performance of the biomarkers in detecting average alcohol consumption of more than 40 g/day for MCV (sensitivity 5%, specificity 99%) and GGT (sensitivity 24%, specificity 87%) were similar to those observed in Izhevsk [16]. The World Health Organization–International Society for Biomedical Research on Alcoholism International Society for Biomedical Research on Alcoholism (WHO–ISBRA) Collaborative Study, a cross-sectional study of 1863 participants in five countries (Australia, Brazil, Canada, Finland and Japan) using convenience samples, found that among the 1250 men, CDT (92% specificity, 60% sensitivity) had a higher specificity for high-risk drinking (>80 g/day) than GGT (74% specificity, 67% sensitivity). The ROC analysis performances of CDT, GGT and AST for detecting average alcohol consumption of more than 40 g/day were similar to those observed in Izhevsk [17].

Our results are unlikely to be due to chance: the associations were strong and consistent.

The extent to which our results can be generalized to Russia as a whole needs to be considered. Sensitivity and specificity are regarded generally as being independent of the prevalence of the outcome. To this extent, whether the population of Izhevsk drink more or less heavily or hazardously does not pose a problem to generalizing our findings. The similarity of our results with those from other populations, many of which have very different patterns and levels of drinking, would support this. We should consider whether the subjects with biomarkers available for analysis are a selected subgroup. However, there was no evidence of any difference in alcohol consumption between those who were included in the analysis and those not, in terms of frequency of consumption (*P* = 0.51) or annual volume of alcohol consumption (*P* = 0.99).

The study was designed to minimize the risk of misclassification and information bias in measuring alcohol consumption [21]. To calculate the annual volume of alcohol consumption, detailed information was required on frequency of consumption and the usual amount consumed. Assumptions were made about the typical alcohol content of each type of drink, and each participant's drinking behaviours was assumed to have been consistent over the questionnaire reference period of the previous 12 months [22]. The questionnaire was validated by comparison of 1564 pairs of subject and proxy answers regarding frequency of alcohol consumption, which found a weighted Cohen's kappa coefficient of 0.61, indicating moderate agreement [21]. Thus, any major source of exposure misclassification would have to affect subjects' and proxies' responses similarly. Like every other study which aims to look at the sensitivity and specificity of biomarkers for alcohol consumption, we do not have a gold standard. It is almost certain that there will be an element of misclassification of men's drinking behaviour, although we have attempted to minimize this, which will lead to the biomarkers having an estimated sensitivity and specificity which may be lower than they really are if judged against a true gold standard. It would be expected that under-reporting of alcohol consumption, if differential, would be greatest among the heaviest drinkers, which would tend rather to underestimate the associations between alcohol intake and biomarker elevation. Non-differential measurement error may well be present among results which rely upon absolute values of alcohol consumption (prevalence of high-risk drinking, and the ‘95% reference ranges’ of biomarkers among men reporting low-volume drinking).

When adjustments were made for age, obesity (waist : hip ratio and BMI), socio-economic status, education and hepatitis B and C status, the odds ratios were largely unchanged, suggesting a minimal confounding effect of these a priori confounders [16,17,20]. Unmeasured confounders could include medications and hepatobiliary disease, which elevate GGT, ALT and AST, and may reduce alcohol consumption [14,17,20].

This study is cross-sectional and does not consider previous or cumulative alcohol exposure. The Russian population alcohol consumption has varied markedly over time [4]. If those suffering health effects of previous heavy drinking tend to stop drinking or reduce the amount they drink, a high proportion of biomarker elevation among light and non-drinkers may be due to previous alcohol consumption, underestimating the association between alcohol and biomarkers when alcohol exposure is assessed through current drinking alone. Those heavy drinkers most susceptible to the pathological effects of alcohol may have died or stopped drinking, and be under-represented among drinkers in the sample. The population of light drinkers will still include those who are most susceptible to alcohol. This survivor bias will tend to underestimate the association of alcohol with biomarkers. The biomarkers in this study are not used clinically to identify light drinkers [14]. Previous and cumulative alcohol exposure may partly explain elevated biomarkers among current drinkers not reporting current risky drinking.

This study has compelling strengths. It is the largest study of the association between alcohol consumption and biomarker levels in the Russian male population, and included a wide range of standard biomarkers. The novel methodology for measuring alcohol consumption could overcome previous difficulties with self-reporting by assessing more objective behavioural indicators of hazardous alcohol consumption. The results suggested that selection bias was minimal. The relationships between alcohol consumption observed were strong, internally consistent and similar to those observed previously in two large cross-sectional studies in other populations.

It is known that hazardous drinking patterns are associated strongly with morbidity and mortality independently of the amount of alcohol consumed [3,6,7]. However, the performance of individual biomarkers for distinguishing hazardous drinking patterns or heavy drinking (rather than current drinking) is poor. Further work could explore combining the present biomarkers and new ones (e.g. phosphatidylethanol) [34,35] as objective indicators of alcohol exposure to determine if this performance can be improved.

## Supporting information

**Table S1** Univariable analysis of the association between possible confounding factors and biomarkers in the top quintile. **Table S2** Sensitivity and specificity of combinations of standard thresholds of gamma-glutamyltransferase (GGT) and carbohydrate-deficient transferrin (CDT) for drinking patterns among participants with a result available for both (*n* = 997). **Table S3** Odds ratios of combinations of gammaglutamyltransferase (GGT) and/or carbohydrate-deficient transferrin (CDT) biomarkers being in the top quintile for the sample among alcohol drinkers excluding surrogate drinkers.
