# Supplementary material for: Comparative performance of biomarkers of alcohol consumption in a population sample of working-aged men in Russia: the Izhevsk Family Study
Source: Addiction. 2013 Jul 5;108(9):1579–89. doi: 10.1111/add.12251 (PMC3992900; doi:10.1111/add.12251)
Supplement: Supplementary file 1 — Table S1 Univariable analysis of the association between possible confounding factors and biomarkers in the top quintile. Table S2 Sensitivity and specificity of combinations of standard thresholds of gamma-glutamyltransferase (GGT) and carbohydrate-deficient transferrin (CDT) for drinking patterns among participants with a result available for both (n = 997). Table S3 Odds ratios of combinations of gammaglutamyltransferase (GGT) and/or carbohydrate-deficient transferrin (CDT) biomarkers being in the top quintile for the sample among alcohol drinkers excluding surrogate drinkers. [file add0108-1579-sd1.docx]

**Supplementary table 1: Univariable analysis of the association between possible confounding factors and biomarkers in the top quintile**

|  | **Total^1^** | **GGT** | | **AST** | | **ALT** | | **CDT** | | **MCV** | |
| --- | --- | --- | --- | --- | --- | --- | --- | --- | --- | --- | --- |
|  |  | **n** | **%** | **n** | **%** | **n** | **%** | **n** | **%** | **n** | **%** |
| **Age at interview** (years) | | | | | | | |  | | | |
| 27-34 | 87 | 16 | 18.4 | 27 | 31.0 | 29 | 33.3 | 24/87 | 27.6 | 18/87 | 20.7 |
| 35-39 | 98 | 23 | 23.5 | 20 | 20.4 | 28 | 28.6 | 16/97 | 16.5 | 12/98 | 12.2 |
| 40-44 | 119 | 29 | 24.4 | 30 | 25.2 | 30 | 25.2 | 29/117 | 24.8 | 21/118 | 17.8 |
| 45-49 | 198 | 37 | 18.7 | 35 | 17.7 | 41 | 20.7 | 31/195 | 15.9 | 35/198 | 17.7 |
| 50-54 | 252 | 53 | 21.0 | 45 | 17.9 | 37 | 14.7 | 50/238 | 21.0 | 45/252 | 17.9 |
| 55-59 | 269 | 49 | 18.2 | 53 | 19.7 | 41 | 15.2 | 56/263 | 21.3 | 77/268 | 28.7 |
| P (Χ^2^) |  | 0.68 | | 0.09 | | <0.001 | | 0.18 | | 0.003 | |
| **Socio-economic status** (car or central heating ownership) | | | | | | | |  | | | |
| Owns neither | 61 | 11 | 18.0 | 16 | 26.2 | 16 | 26.2 | 14/59 | 23.7 | 14/61 | 23.0 |
| Owns either | 473 | 105 | 22.2 | 117 | 24.7 | 104 | 22.0 | 115/460 | 25.0 | 116/472 | 24.6 |
| Owns both | 489 | 91 | 18.6 | 77 | 15.8 | 86 | 17.6 | 77/478 | 16.1 | 78/488 | 16.0 |
| P (Χ^2^) |  | 0.35 | | 0.001 | | 0.11 | | 0.003 | | 0.004 | |
| **Education** (secondary level) | | | | | | | |  | | | |
| Incomplete or lower | 45 | 11 | 24.4 | 11 | 24.4 | 6 | 13.3 | 14/43 | 32.6 | 14/45 | 31.1 |
| Secondary | 748 | 143 | 19.1 | 156 | 20.9 | 144 | 19.3 | 168/728 | 23.1 | 162/746 | 21.7 |
| Higher | 230 | 53 | 23.0 | 43 | 18.7 | 56 | 24.4 | 24/226 | 10.6 | 32/230 | 13.9 |
| P (Χ^2^) |  |  | 0.33 |  | 0.62 |  | 0.12 |  | <0.001 |  | 0.007 |
| **Body mass index, BMI** (kg/m^2^) | | | | | | | |  | | | |
| Normal | 426 | 80 | 18.8 | 97 | 22.8 | 59 | 13.9 | 135/416 | 32.5 | 115/424 | 27.1 |
| Overweight | 406 | 79 | 19.5 | 69 | 17.0 | 77 | 19.0 | 62/398 | 15.6 | 65/406 | 16.0 |
| Obese | 146 | 33 | 22.6 | 31 | 21.2 | 51 | 34.9 | 8/141 | 5.7 | 24/146 | 16.4 |
| Severely obese | 39 | 15 | 38.5 | 11 | 28.2 | 18 | 46.2 | 0/37 | 0 | 3/39 | 7.7 |
| P (Χ^2^) |  | 0.27 | | 0.12 | | <0.001 | | <0.001 | | <0.001 | |
| **Waist:hip ratio** (quartiles) | | | | | | | |  | | | |
| Lowest | 257 | 33 | 12.8 | 57 | 22.1 | 35 | 13.6 | 71/254 | 28.0 | 61/256 | 23.8 |
| Second | 259 | 42 | 16.2 | 43 | 16.6 | 39 | 15.1 | 63/251 | 25.1 | 48/258 | 18.6 |
| Third | 256 | 70 | 26.6 | 50 | 19.5 | 61 | 23.8 | 38/248 | 15.3 | 54/256 | 21.9 |
| Highest | 249 | 64 | 25.7 | 60 | 24.1 | 71 | 28.5 | 34/243 | 14.0 | 45/249 | 18.1 |
| P (Χ^2^) |  | <0.001 | | 0.17 | | <0.001 | | <0.001 | | 0.35 | |
| **Hepatitis B status** (surface antigen) | | | | | | | |  | | | |
| Negative | 909 | 178 | 19.6 | 169 | 18.6 | 174 | 19.1 | 173/887 | 19.5 | 174/908 | 19.2 |
| Positive | 39 | 8 | 20.5 | 10 | 25.6 | 9 | 23.1 | 8/37 | 21.6 | 12/39 | 30.8 |
| Inconclusive | 19 | 6 | 31.6 | 8 | 42.1 | 8 | 42.1 | 6/18 | 33.3 | 5/19 | 26.3 |
| P (Χ^2^) |  | 0.43 | | 0.02 | | 0.04 | | 0.33 | | 0.16 | |
| **Hepatitis C status** (antibody) | | | | | | | |  | | | |
| Negative | 930 | 181 | 19.5 | 169 | 18.2 | 172 | 18.5 | 180/905 | 19.9 | 188/929 | 20.2 |
| Positive | 35 | 10 | 28.6 | 18 | 51.4 | 19 | 54.3 | 7/35 | 20.0 | 2/35 | 5.7 |
| Inconclusive | 2 | 1 | 50.0 | 0 | 0 | 0 | 0 | 0/2 | 0 | 1/2 | 50.0 |
| P (Χ^2^) |  | 0.23 | | <0.001 | | <0.001 | | 0.78 | | 0.06 | |

1. Number of participants with any results available for GGT, ALT and AST (denominators for CDT and MCV results shown in relevant columns)

**Supplementary table 2: Sensitivity and specificity of combinations of standard thresholds of** **gamma-glutamyltransferase (GGT) and carbohydrate-deficient transferrin (CDT) for drinking patterns among participants with a result available for both (n=997)**

|  | **GGT and CDT^1^** | | | **GGT or CDT^2^** | | | **CDT given elevated GGT^3^** | | |
| --- | --- | --- | --- | --- | --- | --- | --- | --- | --- |
|  | **Biomarkers elevated** | **Sensitivity** | **Specificity** | **Biomarker elevated** | **Sensitivity** | **Specificity** | **Biomarker elevated** | **Sensitivity** | **Specificity** |
|  |  | **(%)** | **(%)** |  | **(%)** | **(%)** |  | **(%)** | **(%)** |
| Current drinker | 102/866 | 11.8 | 99.2 | 436/866 | 50.4 | 95.4 | 102/205 | 50.0 | 83.3 |
| Not current | 1/131 |  |  | 6/131 |  |  | 1/6 |  |  |
| Hazardous drinker | 20/94 | 21.3 | 90.9 | 61/94 | 64.9 | 58.2 | 20/29 | 69.0 | 54.5 |
| Not hazardous | 81/888 |  |  | 371/888 |  |  | 81/178 |  |  |
| > 40 g/day | 24/122 | 19.7 | 91.2 | 93/122 | 76.2 | 60.3 | 24/36 | 67.0 | 55.3 |
| < 40 g/day | 76/862 |  |  | 342/862 |  |  | 76/170 |  |  |
| Risky drinker | 34/175 | 19.4 | 91.6 | 121/175 | 69.1 | 61.0 | 34/53 | 64.2 | 56.3 |
| Not risky drinker | 69/820 |  |  | 320/820 |  |  | 69/158 |  |  |

1. Both gamma-glutamyltransferase (GGT) and carbohydrate-deficient transferrin (CDT) elevated above the standard thresholds

2. Either gamma-glutamyltransferase (GGT) or carbohydrate-deficient transferrin (CDT) elevated above the standard thresholds

3. Carbohydrate-deficient transferrin (CDT) among participants with gamma-glutamyltransferase (GGT) elevated above the standard threshold

**Supplementary table 3: Odds ratios of combinations of GGT and/ or CDT biomarkers being in the top quintile for the sample among alcohol drinkers excluding surrogate drinkers**

|  | **GGT and CDT**  n=765 | | | | **GGT or CDT**  n=765 | | | **CDT given GGT is elevated**  n=163 | | |
| --- | --- | --- | --- | --- | --- | --- | --- | --- | --- | --- |
|  | **n** | | **OR^1^** | **95% CI^2^** | **n** | **OR^1^** | **95% CI^2^** | **n** | **OR^1^** | **95% CI^2^** |
| **Total amount of alcohol from beverages per year** (L/year) among current drinkers, adjusted for age | | | | | | | | | | |
| >0 and <2 | 167 | | 1 | - | 167 | 1 | - | 18 | 1 | - |
| 2-4 | 184 | | 1.12 | 0.33 – 3.77 | 184 | 1.79 | 1.07 – 2.99 | 33 | 0.62 | 0.16 – 2.49 |
| 5-9 | 176 | | 3.89 | 1.40 – 10.81 | 176 | 3.47 | 2.11 – 5.71 | 49 | 1.56 | 0.47 – 5.20 |
| 10-19 | 142 | | 3.51 | 1.21 – 10.18 | 142 | 3.22 | 1.91 – 5.42 | 37 | 1.44 | 0.41 – 5.02 |
| 20 + | 96 | | 4.80 | 1.62 – 14.23 | 96 | 7.11 | 4.00 – 12.63 | 26 | 2.48 | 0.65 – 9.42 |
| P ^3^ |  | | 0.001 | |  | <0.0001 | |  | 0.19 | |
| **Total amount of alcohol from beverages per year** (L/year) among current drinkers, adjusted for BMI, waist/ hip ratio, age, socio-economic status, education, Hepatitis B and C status | | | | | | | | | | |
| >0 and <2 |  | | 1 | - |  | 1 | - |  | 1 | - |
| 2-4 |  | | 1.36 | 0.38 – 4.89 |  | 1.92 | 1.13 – 3.26 |  | 1.16 | 0.16 – 8.37 |
| 5-9 |  | | 4.85 | 1.60 – 14.67 |  | 3.71 | 2.22 – 6.21 |  | 1.82 | 0.31 – 10.78 |
| 10-19 |  | | 4.15 | 1.31 – 13.17 |  | 3.26 | 1.90 – 5.60 |  | 1.39 | 0.24 – 8.02 |
| 20 + |  | | 4.58 | 1.44 – 14.63 |  | 7.41 | 4.08 – 13.45 |  | 4.97 | 0.73 – 33.86 |
| P ^3^ | 0.003 | | | | <0.0001 | | |  | 1.00 | |
| P (linearity) ^4^ | | 0.01 | | | <0.0001 | | |  |  | |

1. Odds ratio (OR)

2. 95% confidence interval

3. Likelihood ratio test (LRT) for total amount of alcohol

4. Likelihood ratio test (LRT) for linearity
